# Untargeted serum metabolomics in systemic lupus erythematosus reveals candidate metabolomic signatures associated with clinical phenotypes and glucocorticoid use

**DOI:** 10.3389/fimmu.2026.1925827

**Published:** 2026-10-06

**Authors:** Laura E. Eades, Christopher K. Barlow, Katherine J. Jeppe, Hossein Valipour Kahrood, Ralf B. Schittenhelm, Joanna R. Kent, Arthur Richard Kitching, Artemis Papadaki, William A. Figgett, Rangi Kandane-Rathnayake, Alberta Y. Hoi, Eric F. Morand, Fabien B. Vincent

**Affiliations:** 1 Centre for Inflammatory Diseases, Department of Medicine, School of Clinical Sciences at Monash Health, Monash University, Clayton, VIC, Australia; 2 Monash Proteomics and Metabolomics Platform, Monash Biomedicine Discovery Institute and Department of Biochemistry and Molecular Biology, Monash University, Clayton, VIC, Australia; 3 Department of Nephrology, Monash Health, Clayton, VIC, Australia; 4 National Heart and Lung Institute, Faculty of Medicine, Imperial College London, London, United Kingdom; 5 Data Science Platform, Garvan Institute of Medical Research, Darlinghurst, NSW, Australia

**Keywords:** biomarkers, glucocorticoids, lupus nephritis, lysine, metabolomics, remission, systemic lupus erythematosus

## Abstract

**Objectives:**

Despite promising advances in metabolomics for biomarker discovery in systemic lupus erythematosus (SLE), studies examining associations with detailed disease phenotypes, including organ damage and validated outcome measures, remain limited. We aimed to explore associations between serum metabolomic profiles with SLE phenotypes.

**Methods:**

Untargeted serum metabolomics was performed in 60 SLE patients using liquid chromatography-mass spectrometry. Metabolites with missing values or low-confidence assignments were excluded. Differential abundance analysis (DA) using regression models and supervised learning using least absolute shrinkage and selection operator (LASSO) were performed to investigate relationships between metabolites and clinical features. Covariates in multivariable models included age, sex, ethnicity and renal impairment (eGFR < 60 mL/min/m2). Disease activity was assessed using the SLE Disease Activity Index-2000 (SLEDAI-2K), with renal activity defined as the presence of SLEDAI-2K proteinuria.

**Results:**

364 metabolites were studied in 58 patients (median (IQR) age 42 (34-52) years, 91% female, 50% Asian ethnicity, 50% European ethnicity, 50% active disease). Using DA, 20 metabolites were significantly associated with glucocorticoid use, although no predictive LASSO model for this feature was identified. A metabolomic feature putatively identified as 5β-Cholestane-3α, 7α, 24, 25-tetrol was significantly negatively associated with biopsy-proven lupus nephritis (LN) and active LN. Glutamic acid was significantly positively associated with glucocorticoid-free remission. Homocitrulline and N6, N6, N6-trimethyl-L-lysine were significantly positively associated with active LN. A feature putatively identified as 3-Hydroxy-N6, N6, N6-trimethyl-L-lysine was significantly positively associated with biopsy-proven LN. These metabolite associations were also predictive on LASSO modelling. Pathway-level enrichment revealed associations with active disease (Valine, Leucine and Isoleucine Degradation, FDR = 0.0015), organ damage (Citric Acid Cycle and Gluconeogenesis, FDR = 0.043 each), DORIS remission (Phenylalanine and Tyrosine Metabolism, FDR = 0.028; Valine, Leucine and Isoleucine Degradation, FDR = 0.041), and remission off prednisolone (Urea Cycle, Tryptophan Metabolism, Purine Metabolism, and Glutamate Metabolism; FDR = 0.044 each).

**Conclusions:**

Associations between glucocorticoid use and serum metabolites highlight the importance of accounting for this in SLE metabolomics studies. Significant associations between serum metabolites and SLE phenotypes, particularly previously unreported associations between methylated and carbamylated lysine derivatives with LN, support the promise of serum metabolomics to identify prognostic SLE biomarkers.

## Introduction

Systemic lupus erythematosus (SLE) is a heterogeneous, multisystem inflammatory autoimmune disease associated with significant morbidity and mortality (1), with most patients accruing irreversible organ damage during the course of their disease (1). SLE typically affects women, and is more prevalent and severe in peoples of non-European ethnicity, including peoples of African, Asian and First Nations ethnicities (2). Due to its heterogeneous nature, disease activity and treatment response are inherently difficult to measure accurately (1). This has negatively impacted many SLE clinical trials, resulting in a paucity of new targeted treatments being approved, despite major progresses in some other autoimmune rheumatic diseases such as rheumatoid arthritis (RA) (3). The discovery of biomarkers, which accurately reflect SLE disease activity and treatment response could play a pivotal role in improving clinical trial outcomes, ultimately leading to the development of improved targeted treatments and outcomes for patients.

Metabolomics is the untargeted, large-scale study of metabolites within a biological system; it facilitates the study of thousands of compounds simultaneously, providing a reflection of the biochemical state of this system at a particular point in time (4). As such, metabolomics is emerging as a promising method of biomarker discovery. There are numerous clinically validated metabolite biomarkers in clinical practice, with a widely used example being serum creatinine as a measure of renal function (5). Metabolomics is most commonly conducted using gas chromatography or liquid chromatography-tandem mass spectrometry (LC-MS) or nuclear magnetic resonance spectroscopy, and can be performed on various biological fluids, including urine, faeces, plasma and serum (4).

A number of studies of serum or plasma metabolomics in SLE have identified promising biomarkers (4). Patients with SLE and those with other autoimmune rheumatic diseases, including RA, Sjögren disease and systemic sclerosis reportedly share features in their metabolite profiles that differ from those of healthy controls, while some metabolite features are reported specific to SLE (6, 7). Furthermore, studies have demonstrated relationships between metabolomic profiles and SLE phenotypes. Recently, Zhang Y et al. performed comprehensive serum metabolomics in SLE patients with and without lupus nephritis (LN) and healthy controls, finding that metabolomic profiles differed significantly between the three groups (8). In particular, the authors identified five metabolites (SM d34:2, DG (18:3(9Z, 12Z, 15Z)/20:5(5Z, 8Z, 11Z, 14Z, 17Z)/0:0), nervonic acid, Cer-NS d27:4, and PC (18:3(6Z, 9Z, 12Z)/18:3(6Z, 9Z, 12Z)) which were able to delineate SLE patients with and without LN using differential abundance analysis (8). More specifically, a study of urinary metabolomics demonstrated that proliferative LN could be distinguished from membranous LN by differing levels of urinary citrate and taurine (9). Other studies have demonstrated distinct groups of metabolites associated with renal, cutaneous, haematological and multi-system involvement in patients with SLE (10, 11), as well as metabolites associated with active and inactive disease (10, 12). Moreover, some medications used to treat SLE, including anifrolumab and voclosporin, have been shown to cause alterations in metabolomic and lipidomic profiles, respectively (13, 14).

However, despite these promising findings, there have been few metabolomics studies examining associations between metabolites and detailed clinical SLE phenotypes, including organ damage and validated disease outcome measures such as Lupus Low Disease Activity State (LLDAS) and Definition of Remission in SLE (DORIS) remission. In addition, few studies have explored the impact that widely used SLE medications, such as glucocorticoids, may have on metabolomic profiles in SLE. In this study, we aimed to generate MS-based untargeted serum metabolomic profiles in a multi-ethnic SLE cohort and explore associations with socio-demographics, SLE phenotypes and outcomes.

## Materials and methods

The SLE patient cohort and methodology for this study are similar to our recent studies of serum IgG and IgA autoantibody profiling in SLE (15, 16).

### Study participants

Sixty patients with SLE were included in this retrospective, single-centre, cross-sectional, exploratory, hypothesis-generating study. All study participants were enrolled in the Australian Lupus Registry and Biobank (ALRB) (17), and recruited at the Monash Health Lupus Clinic (Melbourne, Australia) between July 2019 and November 2020. All participants fulfilled either the 1997 American College of Rheumatology (ACR) revised criteria or the Systemic Lupus International Collaborating Clinics (SLICC) criteria for SLE classification (18, 19). Careful participant selection, guided by a purposive sampling approach, meant that the study cohort included equal proportions of individuals of self-identified European and Asian ethnicity, and was balanced in terms of age, sex and disease activity, as per SLE Disease Activity Index-2000 (SLEDAI-2K) (20).

All participants gave informed written consent for their stored clinical data and biosamples to be used in SLE-related research. Ethics approval was granted by the Monash Health Human Research Ethics Committee (Ref: 13019A). This study was performed in accordance with the National Statement on Ethical Conduct in Human Research (2007).

### Data collection & clinical assessment

Baseline socio-demographic data, including date of birth, sex, self-reported ethnicity, employment, household income level, education level, smoking status and alcohol consumption were extracted from the ALRB database. Cross-sectional clinical data were obtained from the ALRB clinic visit closest to the date of study serum sample collection (within 30 days), including disease activity, flare, routine laboratory tests and medication use (prednisolone, hydroxychloroquine, immunosuppressants). For data regarding organ damage, the annual visit closest to study sample collection was used. Disease activity was assessed using the SLEDAI-2K (20); active disease was defined as SLEDAI-2K > 4 (21). Active LN was defined as the presence of SLEDAI-2K proteinuria (urine protein:creatinine ratio > 0.05 g/mmol); haematuria or pyuria in the absence of proteinuria were not considered active LN in this study, as they are non-specific findings in isolation. Organ damage was assessed annually using the SLICC-ACR Damage Index (SDI) (22); SDI > 0 was defined as the presence of organ damage. Low disease activity was defined as meeting criteria for LLDAS (23), and clinical remission was defined as meeting criteria for DORIS (24). DORIS remission without glucocorticoid use was also studied as a more stringent definition of remission. Flare was defined using the SELENA-SLEDAI flare index (SFI) (25). Extracted laboratory data included the results of any previous kidney biopsy, as well as erythrocyte sedimentation rate (ESR) and estimated glomerular filtration rate (eGFR) on the day of study serum sample collection. Renal impairment was defined as an eGFR < 60 ml/min/m2. A more detailed list of clinical data extracted is provided in **Supplementary Table 1**.

### Biological sample collection, processing & storage

Metabolomics analysis was performed on a single serum sample, collected via venepuncture between July 2019 and November 2020, from each study participant. Blood collection, processing and serum storage was performed as described previously (26).

### Serum metabolomics

Samples were prepared for LC-MS analysis by treating 25 µL of serum with 200 µL of 1:1 acetonitrile:methanol that had been chilled on ice. The samples were then shaken on a vortex mixture for 20 minutes at 4 °C before being centrifuged at 22, 000 × g for 10 minutes at 4 °C. 180 μl of the resulting supernatant were transferred to an LC-MS vial for LC-MS analysis. A pooled quality control (PQC) sample was prepared by combining a small equal volume from each extract.

For LC-MS analysis, samples were analyzed by hydrophilic interaction LC (HILIC) coupled to high-resolution MS (LC-MS) (27). In brief, we utilized a polymeric ZIC-pHILIC, 20 x 2.1 mm guard column coupled to a ZIC-pHILIC 5 µm, 150 x 4.6 mm analytical column (Merck, Australia) at 25 °C with a gradient elution of 20 mM ammonium carbonate (A) and acetonitrile (B) (linear gradient time-%B as follows: 0 min-80%, 15 min-50%, 18 min-5%, 21 min-5%, 24 min-80%, 32 min-80%) on a Dionex RSLC3000 UHPLC (Thermo). The flow rate was maintained at 300 µL/min. Samples were kept at 6 °C in the autosampler and 10 µL injected for analysis. MS was performed on a Q-Exactive Plus Orbitrap (Thermo Fisher Scientific, Australia) with all samples analysed in MS1 polarity switching mode. The instrument was operated at a resolution of 70, 000 with the following conditions: Electrospray ionization voltage was 4 kV in positive and -3.5 kV in negative mode, capillary temperature = 300 °C; sheath gas = 50; Aux gas = 20; probe temp = 120 °C). All samples were analysed in randomized order and with PQC samples analysed every seventh sample throughout the batch to confirm reproducibility. Additionally, the PQC sample was also analysed in data dependent acquisition mode (DDA) with separate injections for positive and negative mode. DDA was performed with the following parameters: TopN = 5; dynamic exclusions 10s; isolation window 2.0 m/z; (N)CE = 20, 30, 40 and resolution of 70, 000 and 35, 000 for MS1 and MS2 scans respectively. MS1 LC-MS data was processed in an untargeted fashion using opensource software IDEOM (28), which initially used msConvert (ProteoWizard) (29) to convert raw LC-MS files to mzXML format and XCMS to pick peaks to convert to.peakML files (30). Mzmatch was subsequently used for sample alignment and filtering.

A standard library containing ~ 450 metabolites was analysed alongside the samples to allow direct comparison of the retention times of those compounds as well as forming the basis of a retention time prediction model used to provide putative identification of metabolites not contained within the standard library (31). Of note, not all metabolites in the standard library were detectable in our patient cohort. Metabolomic features that were not directly supported by the standard library have been described as putative in text.

### Statistical & bioinformatics analyses

Data analyses and visualisation were performed using R (version 4.4.0) and R Studio version (2024.12.1) software. A *p*-value of < 0.05 was considered statistically significant. All reported *p*-values were adjusted for multiple comparisons using the Bonferroni correction or the Benjamini–Hochberg procedure, unless explicitly labelled as nominal *p*.

#### Data filtering and missing data

Metabolites for which there were any missing values for metabolite abundance across samples were excluded from analysis. This filtering approach reduced data dimensionality, limiting overfitting and mitigating the curse of dimensionality in downstream analyses. Patients were excluded from analysis of a particular clinical variable if they had missing data for that clinical variable (maximum number of patients excluded for any variable was n = 4).

To focus on high-confidence metabolites, a high identity confidence dataset was selected for downstream analysis, meaning that all metabolites with an IDEOM identity confidence score < 8 (out of possible 10) were excluded.

#### Data transformation and normalisation

Metabolite abundances were median-normalised to reduce between-sample differences in overall signal intensity, and to reduce the influence of global multiplicative effects, including differences in serum concentration (e.g., hydration status) and technical variation. Median-normalised data were then log_2_-transformed to reduce right-skewness and improve approximation to normality. The resulting metabolite matrix was mean-centred and scaled to unit variance using the scale () function (base R package); this transformation was applied metabolite-wise, so that each metabolite had a mean of zero and a standard deviation of one across samples, allowing metabolites measured on different abundance scales to contribute comparably to downstream analyses.

#### Descriptive statistics

Categorical variables are reported as frequency (percentage). Continuous variables are summarised as mean [standard deviation (SD)] for normally distributed variables and median [interquartile range (IQR)] for non-normally distributed variables.

#### Heatmaps and outlier assessment

Heatmaps with hierarchical clustering depicting scaled and centred metabolite abundance across the patient cohort were created using the Heatmap() function from the ComplexHeatmap R package (version 2.20.0). To assess for outliers in the patient cohort, principal component analysis (PCA) was performed using the PCA() function in the FactoMineR package (version 2.11). Using visual inspection of PCA plots, created using the fviz_pca_ind() function (factoextra R package; version 1.0.7), potential patient outliers were identified. LC-MS experimental quality control data and heatmaps were reviewed to determine whether technical issues may have contributed to outlier status. Clinical data were also reviewed to assess for the presence of patient-specific characteristics which may have significantly impacted on metabolite profiles, leading to outlier status.

#### Unsupervised clustering and association analysis

Using the median-normalised, log_2_-transformed metabolite abundance matrix that was centred and scaled, samples were clustered in an unsupervised manner based on metabolite profiles. PCA was used for exploratory visualisation of the global data structure. The contribution of individual metabolites to the principal components was quantified using squared cosine (cos²) values for PC1 and PC2.

The optimal number of clusters (k) was evaluated, independently of PCA, using multiple complementary approaches, including within-cluster sum of squares (elbow method), the gap statistic (Kmax = 10; B = 50 permutations), average silhouette width, and consensus clustering. Consensus clustering was performed with ConsensusClusterPlus (maxK = 10, reps = 1000) using hierarchical clustering with Euclidean distance. Based on concordance across these criteria, k = 3 was selected for downstream analyses. Final cluster assignments were derived using agglomerative hierarchical clustering with Euclidean distance and complete linkage, with the dendrogram cut at k = 3.

Associations between cluster membership (outcome) and clinical features (exposure) were explored using univariable multinomial logistic regression. Multivariable models were not fitted due to small sample size in some clusters limiting the ability to adjust for covariates. Each cluster was used in turn as the reference cluster in the regression models, meaning that all clusters were directly compared to each other. For clinical features of interest in clusters where multinomial logistic regression could not be fitted due to a complete separation case, bivariate analysis was performed using Fisher’s exact tests adjusted for multiple testing for each particular clinical variable of interest (Bonferroni correction).

#### Differential abundance analysis

Associations between individual metabolites (outcome) and clinical features (exposure) were assessed via differential abundance analysis (DA), using both univariable and multivariable linear regression models. Multivariable models included adjustment for age, sex, ethnicity, and renal impairment. A sensitivity analysis adjusting for glucocorticoid use in addition to the aforementioned covariates was also performed. For all DA linear regression models, a minimum of n = 12 patients were permitted in a subgroup, and the Benjamini-Hochberg adjustment for multiple testing was applied.

#### Predictive modelling

Supervised modelling using least absolute shrinkage and selection operator (LASSO) regression was performed to create parsimonious metabolite-based models which could potentially be used to predict each clinical feature of interest. A separate LASSO model was fitted for each categorical clinical feature of interest. Patient age, sex, ethnicity and renal impairment were included as covariates in the LASSO models alongside all metabolites. Metabolite abundances were included in the LASSO models after preprocessing as described above. Other covariates were similarly prepared for model fitting, including encoding and scaling/centring where applicable, to ensure that all predictors were appropriately represented before penalisation. LASSO models were implemented using the train() and trainControl() functions from the caret R package (version 7.0-1), with the glmnet method. Model tuning was performed using repeated 6-fold cross-validation with 100 repeats. The optimal penalty parameter, lambda (λ), was selected according to the cross-validated area under the receiver operating characteristic curve (ROC AUC). Model performance was assessed using overall classification accuracy; accuracy of at least 0.7 was required for a model to be deemed satisfactory, and the results reported.

#### Metabolite set enrichment analysis

To assess coordinated pathway-level metabolic changes, rank-based metabolite set enrichment analysis (MSEA) was performed for each clinical feature of interest using the fgsea R package. A rank-based approach was chosen over threshold-based over-representation analysis, as it uses the full ranked metabolite distribution rather than an arbitrary significance cutoff, enabling detection of coordinated but individually modest metabolic associations. For each clinical feature, all 364 metabolites were ranked by their moderated t-statistic (which incorporates both magnitude and direction of association). Metabolite identifiers were mapped to the Small Molecule Pathway Database (SMPDB) via MetaboAnalystR (v4.3.0). Enrichment was tested against SMPDB metabolic pathways (5–200 members), with pathway-level significance defined as FDR (Benjamini-Hochberg) < 0.05, corrected within each clinical feature’s set of tested pathways.

## Results

### SLE cohort characteristics

1, 899 serum metabolites were detected in 60 patients with SLE. Following exclusion of metabolites with missing values, and those with an IDEOM metabolite identity confidence score of < 8 (out of possible 10), 364 metabolites were retained for downstream analyses, including clustering, DA and LASSO. Three potential patient outliers were identified using PCA (**Figure 1A**). Upon review of clinical data, heatmaps of serum metabolite abundance matrix and LC-MS quality control data, one potential patient outlier was excluded (end-stage kidney failure, deemed likely to substantially influence the patient’s metabolomic profile); additionally, another sample (not an outlier on PCA) was excluded due to a change in LC-MS instrument performance identified immediately prior to acquisition. There was no indication of any significant clinical or technical factors affecting the two other potential PCA-defined patient outliers; therefore, these samples were retained for downstream analyses.

**Figure 1:**
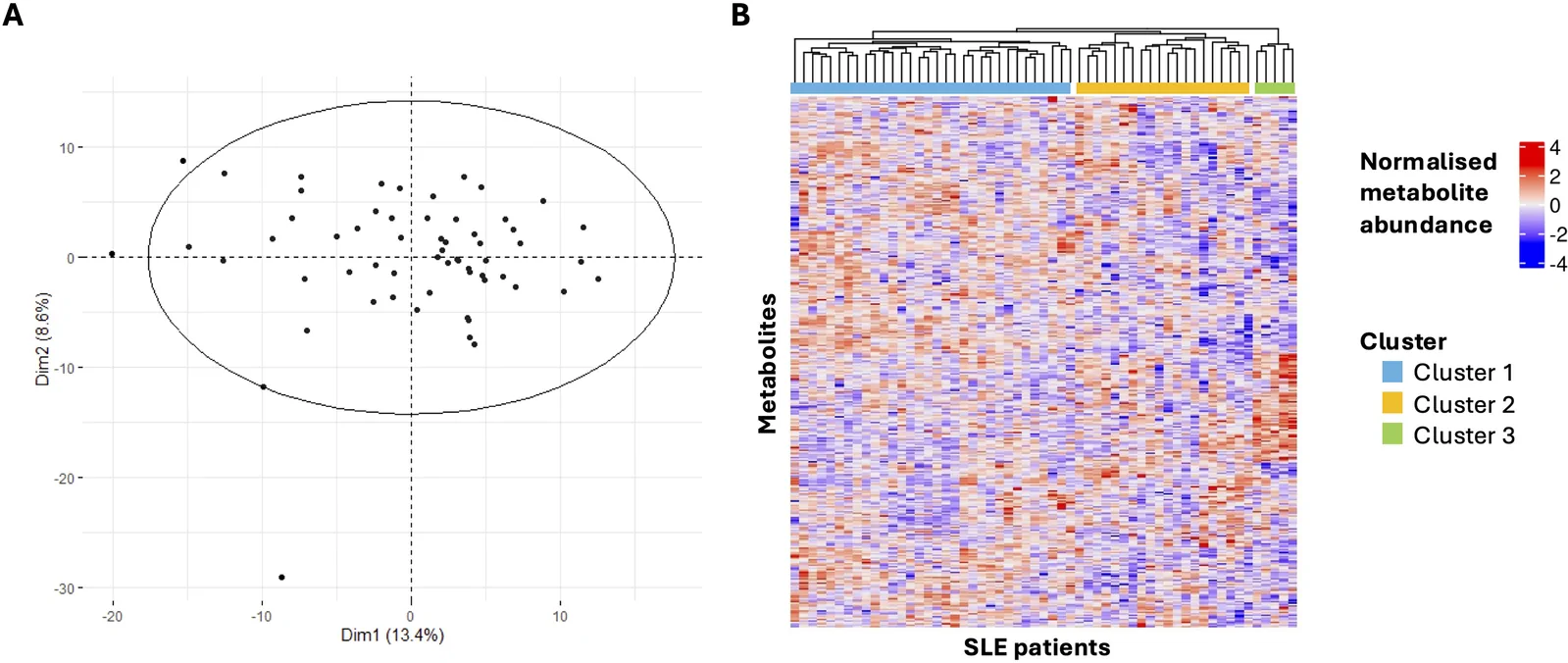
Hierarchical clustering of patients with SLE according to serum metabolomic profiles. **(A)** Principal component analysis (PCA) plot of patient samples (N = 60) projected onto the first two principal components. Samples located outside the confidence ellipse are considered potential outliers. **(B)** Heatmap showing hierarchical clustering of scaled and centred normalised serum metabolite abundances (rows) across SLE patients (columns). Metabolite abundance level is color-coded in with dark blue and red colours indicating the lowest and highest values, respectively. The top annotation bar indicates the patient cluster assignment derived from unsupervised clustering (Cluster 1: n = 32; Cluster 2: n = 20; Cluster 3: n = 5).

The final cohort analysed comprised 58 patients with SLE with a median (IQR) age of 42 (34 – 52) years, and a median (IQR) disease duration of 12 (5 – 18) years. Consistent with the purposive sampling framework underpinning the study, key characteristics were evenly represented across the cohort, particularly with 50% of participants of Asian ethnicity and 50% of European ethnicity, and 50% having active disease at the time of serum sample collection. Overall, 91% of the cohort was female, and 61% had irreversible organ damage in at least one SDI domain. A more detailed summary of cohort characteristics is shown in **Table 1**.

**Table 1:** Demographics and clinical characteristics of the SLE cohort.

Characteristics	SLE (N = 58)
Age (years)*, median (IQR)*	42 (34-52)
Female sex*, n (%)*	53 (91.4%)
Asian ethnicity^*^, n (%)	29 (50.0%)
Paid employment*, n (%)*	24 (41.4%)
Disability pension*, n (%)*	8 (13.8%)
Household income (< AUD$35K pa)*, n (%)*	16 (27.6%)
Education (< secondary level)*, n (%)*	7 (12.1%)
Current smoker*, n (%)*	5 (8.6%)
Alcohol (>10 standard drinks/week)*, n (%)*	1 (1.7%)
Disease duration (years)*, median (IQR)*	12.1 (5.1-18.3)
Active disease (SLEDAI-2K > 4)*, n (%)*	29 (50.0%)
Active renal disease (SLEDAI-2K proteinuria > 0)*, n (%)*	17 (29.3%)
Biopsy-proven lupus nephritis*, n (%)*	26 (44.8%)
Highly active disease (SLEDAI-2K ≥ 10)*, n (%)*	9 (15.5%)
Low disease activity (LLDAS)^#^*, n (%)*	21 (36.8%)
Clinical remission (with prednisolone ≤ 5 mg)*, n (%)*	18 (31.0%)
Flare*, n (%)*	9 (15.5%)
Organ damage (SDI > 0)^^^*, n (%)*	33 (61.1%)
Renal impairment (eGFR < 60)*, n (%)*	7 (12.1%)
Current glucocorticoid use*, n (%)*	26 (44.8%)
Current hydroxychloroquine use*, n (%)*	51 (87.9%)
Current immunosuppression use*, n (%)*	44 (75.9%)

^*^All patients not of Asian ethnicity were of European ethnicity.

^#^ Data missing for 1 patient, ^^^ data missing for 4 patients.

eGFR, estimated glomerular filtration rate; IQR (interquartile range), LLDAS (lupus low disease activity state), SDI (Systemic Lupus International Collaborating Clinics/American College of Rheumatology SLE Damage Index), SLE (systemic lupus erythematosus), SLEDAI-2K (SLE Disease Activity Index-2000).

### Unsupervised clustering analysis

Using an unbiased, data-driven approach, patients were grouped into clusters based on their metabolite profiles using hierarchical clustering. During initial hierarchical clustering analysis, a single patient was identified as an outlier, forming a distinct cluster separate from the remainder of the cohort. As this observation was considered likely to disproportionately influence clustering results, this patient was excluded from the clustering analysis, but retained for subsequent supervised analyses. Following exclusion of this patient, k = 3 was selected as the optimal number of clusters, based on concordance across multiple clustering validity metrics (**Figure 1B**). Based on univariable multinomial logistic regression, patients in Cluster 1 were less likely to have organ damage (OR 0.06; 95% CI 0.01, 0.33; nominal *p* = 0.001) and less likely to use prednisolone (OR 0.25; 95% CI 0.07, 0.80; nominal *p* = 0.02) than those in Cluster 2. Patients in Cluster 3 were more likely to have active LN than those in Cluster 1 (OR 14.3; 95% CI 1.37, 150; nominal *p* = 0.026), or Cluster 2 (OR 12; 95% CI 1.07, 134; nominal *p* = 0.044), and were more likely to have renal impairment than those in Cluster 2 (OR 76.1; 95% CI 3.88, 1, 490; nominal *p* = 0.004). For clinical features of interest in clusters where multinomial logistic regression could not be fitted due to a complete separation case, Fisher’s exact tests were performed. Patients in Cluster 3 were more likely to have organ damage (*p* = 0.009) and renal impairment (*p* < 0.001) than those in Cluster 1. Patients in Cluster 3 also had numerically higher rates of biopsy-proven LN and active disease according to SLEDAI-2K compared to those in Clusters 1 and 2 (**Supplementary Table 2**), although these results did not reach statistical significance.

To identify the metabolite features contributing most to this cluster structure, we ranked all metabolites by their combined cos² value for PC1 and PC2. The highest-ranked metabolites (alpha- and beta-D-glucose, imidazoleacetic acid riboside, N-formimino-L-glutamate, 2, 3, 4, 5-tetrahydroxypentanoic acid, 2, 3-dihydroxy-isovalerate, and homocitrulline) were specifically elevated in Cluster 3, rather than varying incrementally across all three clusters (**Supplementary Table 3**).

### Associations between SLE clinical features and metabolites

We next assessed for relationships between each individual metabolite and each clinical feature of interest using DA. Using supervised machine learning (LASSO), we also developed parsimonious metabolite models for each of these clinical features, which could potentially be used to predict their presence in other SLE patient cohorts. We particularly focused on robust features, defined as those that were statistically significant in multivariable DA and independently selected by LASSO modelling.

#### Metabolite associations with ethnicity

Five metabolomic features, identified as ergothioneine (adjusted β 1.04; 95% CI 0.58, 1.50; *p* = 0.013), Homo-L-arginine (adjusted β 0.82; 95% CI 0.38, 1.26; *p* = 0.046) and putatively identified as LysoPC 20:3 (adjusted β -0.86; 95% CI -1.29, -0.42; *p* = 0.044), DG 36:4 (adjusted β 0.87; 95% CI 0.38, 1.35; *p* = 0.0497) and PS O-34:1 (adjusted β -0.83; 95% CI -1.27, -0.38; *p* = 0.046) were associated with Asian ethnicity using multivariable differential abundance analysis (**Table 2**; **Figure 2**). However, no good quality LASSO model for Asian ethnicity was fitted (**Table 3**).

**Table 2:** Associations between serum metabolites with SLE clinical features using differential abundance analysis.

Exposure of interest	Metabolites	Metabolite annotation	Univariable models			Multivariable models ^1^			Multivariable models ^2^		
			β-coef	95% CI	*p*	β-coef	95% CI	*p*	β-coef	95% CI	*p*
Asian ethnicity	Ergothioneine	RT std	1.07	0.63, 1.52	0.004	1.04	0.58, 1.50	0.013	1.01	0.54, 1.48	0.026
	Homo-L-arginine	RT std	0.95	0.48, 1.42	0.026	0.82	0.38, 1.26	0.046	0.87	0.42, 1.32	0.037
	LysoPC 20:3	Putative	–	–	–	-0.86	-1.29, -0.42	0.044	-0.81	-1.25, -0.37	0.37
	PS O-34:1	Putative	–	–	–	-0.83	-1.27, -0.38	0.046	-0.86	-1.31, -0.4	0.037
	DG 36:4	Putative	–	–	–	0.87	0.38, 1.35	0.0497	–	–	–
	Bilirubin	RT std ^4^	–	–	–	–	–	–	0.85	0.39, 1.30	0.037
Active disease (SLEDAI-2K > 4)	–		–	–	–	–	–	–	–	–	–
Active renal disease (SLEDAI-2K proteinuria score > 0)	5β-Cholestane-3α, 7α, 24, 25-tetrol	Putative	-1.45	-1.88, -1.01	<0.001	-1.51	-2.02, -1	<0.001	-1.47	-1.99, -0.95	<0.001
	Homocitrulline	RT std	1.15	0.65, 1.64	0.004	–	–	–	–	–	–
	N6, N6, N6-trimethyl-L-Lysine	RT std	0.96	0.44, 1.49	0.046	–	–	–	–	–	–
	3-Hydroxy-N6, N6, N6-trimethyl-L-lysine	Putative	1.06	0.55, 1.57	0.013				–	–	–
	2, 3-Dihydroxy-isovalerate	Putative	0.93	0.39, 1.45	0.046	–	–	–	–	–	–
	Dodecadienoic acid	Putative	-0.93	-1.46, -0.41	0.046	–	–	–	–	–	–
	Cystine	RT std	0.94	0.42, 1.47	0.046	–	–	–	–	–	–
Biopsy-proven LN	Homocitrulline	RT std	0.94	0.47, 1.41	0.023	0.94	0.49, 1.39	0.022	0.94	0.48, 1.39	0.024
	5β-Cholestane-3α, 7α, 24, 25-tetrol	Putative	-1.25	-1.67, -0.84	<0.001	-1.29	-1.74, -0.85	<0.001	-1.29	-1.72, -0.85	<0.001
	3-Hydroxy-N6, N6, N6-trimethyl-L-lysine	Putative	0.96	0.49, 1.43	0.023	–	–	–	–	–	–
	Sulfate	Putative	0.89	0.42, 1.37	0.039	–	–	–	–	–	–
Organ damage (SDI > 0)	Gamma-Aminobutyric acid	RT std	-0.91	-1.42, -0.4	0.025	–	–	–	–	–	–
	Valine	RT std	-0.82	-1.33, -0.31	0.047	–	–	–	–	–	–
	Methylimidazole acetaldehyde	Putative	-0.92	-1.41, -0.42	0.025	–	–	–	–	–	–
	2-Methylhexanoic acid	Putative	-0.83	-1.36, -0.3	0.049	–	–	–	–	–	–
	Isoleucine	RT std	-0.86	-1.36, -0.36	0.029	–	–	–	–	–	–
	Glutamine	RT std	-1.04	-1.54, -0.55	0.012	–	–	–	–	–	–
	Methionine	RT std	-0.81	-1.31, -0.3	0.047	–	–	–	–	–	–
	Histidine	RT std	-1.06	-1.54, -0.58	0.012	–	–	–	–	–	–
	N-Formimino-L-glutamate	Putative	0.82	0.3, 1.35	0.049	–	–	–	–	–	–
	Homocitrulline	RT std	1.01	0.52, 1.51	0.013	–	–	–	–	–	–
	Galacturonate	Putative	0.91	0.39, 1.43	0.028	–	–	–	–	–	–
	Glucuronate	Putative	0.89	0.37, 1.41	0.029	–	–	–	–	–	–
	2-C-Methyl-D-erythritol 4-phosphate	Putative	-0.97	-1.47, -0.46	0.020	–	–	–	–	–	–
	Tridecadienoic acid	Putative	-0.94	-1.45, -0.42	0.025	–	–	–	–	–	–
	Deoxyuridine	RT std	-0.88	-1.39, -0.37	0.029	–	–	–	–	–	–
	Cystine	RT std	0.79	0.29, 1.29	0.049	–	–	–	–	–	–
	Nonanoic acid	Putative	-0.91	-1.42, -0.39	0.028	–	–	–	–	–	–
	5β-Cholestane-3α, 7α, 24, 25-tetrol	Putative	-1.04	-1.54, -0.54	0.012	–	–	–	–	–	–
	PC 34:2	Putative	-0.86	-1.34, -0.39	0.025	–	–	–	–	–	–
	Thyroxine	RT std	-0.69	-1.1, -0.28	0.034	–	–	–	–	–	–
	TG 55:0	Putative	-1.002	-1.5, -0.51	0.013	–	–	–	–	–	–
LLDAS	-		–	–	–	–	–	–	–	–	–
Clinical remission (with prednisolone ≤ 5 mg)	-		–	–	–	–	–	–	–	–	–
Remission off prednisolone	Glutamic acid	RT std	1.14	0.59, 1.70	0.048	1.2	0.62, 1.78	0.048	–	–	–
Prednisolone use	Serine	RT std	-0.87	-1.35, -0.38	0.032	-0.94	-1.35, -0.53	0.004	N/A	N/A	N/A
	Gamma-aminobutyric acid	RT std	-1.05	-1.51, -0.6	0.004	-1.12	-1.51, -0.72	0.0002	N/A	N/A	N/A
	Deoxyuridine	RT std	-0.87	-1.35, -0.39	0.032	-0.91	-1.38, -0.45	0.016	N/A	N/A	N/A
	2, 3-Dihydroxy-isovalerate	Putative	1.06	0.61, 1.51	0.004	0.95	0.52, 1.37	0.004	N/A	N/A	N/A
	LysoPC 18:0	Putative	-0.84	-1.33, -0.36	0.037	-0.96	-1.43, -0.49	0.010	N/A	N/A	N/A
	5-oxo-D-proline	Putative	-0.91	-1.38, -0.43	0.03	-0.95	-1.37, -0.52	0.004	N/A	N/A	N/A
	2-Keto-6-aminocaproate	Putative	0.93	0.46, 1.40	0.026	0.93	0.42, 1.43	0.030	N/A	N/A	N/A
	Glycyl-glycine	RT std	-0.83	-1.31, -0.34	0.04	-0.83	-1.33, -0.32	0.042	N/A	N/A	N/A
	4-oxoglutaramate	Putative	0.87	0.39, 1.35	0.032	0.81	0.34, 1.28	0.031	N/A	N/A	N/A
	Ectoine	RT std	0.83	0.35, 1.32	0.039	0.76	0.27, 1.24	0.0499	N/A	N/A	N/A
	Dimethylglycine	RT std	0.86	0.38, 1.34	0.032	–	–	–	N/A	N/A	N/A
	Sulfate	Putative	0.81	0.32, 1.30	0.049	–	–	–	N/A	N/A	N/A
	2-Hydroxyhexadecanoic acid	Putative	–	–	–	-0.76	-1.25, -0.28	0.049	N/A	N/A	N/A
	Formyl isoglutamine	Putative	–	–	–	-0.86	-1.37, -0.35	0.032	N/A	N/A	N/A
	PE 35:2	Putative	–	–	–	0.77	0.33, 1.2	0.031	N/A	N/A	N/A
	Bilirubin	RT std	–	–	–	-0.80	-1.26, -0.34	0.031	N/A	N/A	N/A
	Thyroxine	RT std	–	–	–	-0.85	-1.35, -0.36	0.031	N/A	N/A	N/A
	5-Methylcytosine	RT std	–	–	–	-0.79	-1.24, -0.34	0.031	N/A	N/A	N/A
	Glucose	Putative	–	–	–	0.70	0.29, 1.11	0.031	N/A	N/A	N/A
	Uridine	RT std	–	–	–	-0.77	-1.25, -0.29	0.048	N/A	N/A	N/A
	LysoPC 22:5	Putative	–	–	–	-0.81	-1.3, -0.32	0.037	N/A	N/A	N/A
	TG 55:0	Putative	–	–	–	-0.80	-1.26, -0.35	0.031	N/A	N/A	N/A
Immunosuppression use	–		–	–	–	–	–	–	–	–	–

^1^ RT std indicates the metabolite annotation was matched on retention time against a standard, while putative indicates a putative identification.

^2^ adjusted for socio-demographics and renal impairment.

^3^ adjusted for socio-demographics, renal impairment and glucocorticoid use.

^4^ Supported by RT standard but very early eluting.

- No statistically significant results.

CI, confidence interval; LLDAS, lupus low disease activity state; N/A, not applicable; RT std, retention time against a standard; SDI, Systemic Lupus International Collaborating Clinics/American College of Rheumatology SLE Damage Index; SLEDAI-2K, SLE Disease Activity Index-2000.

**Figure 2:**
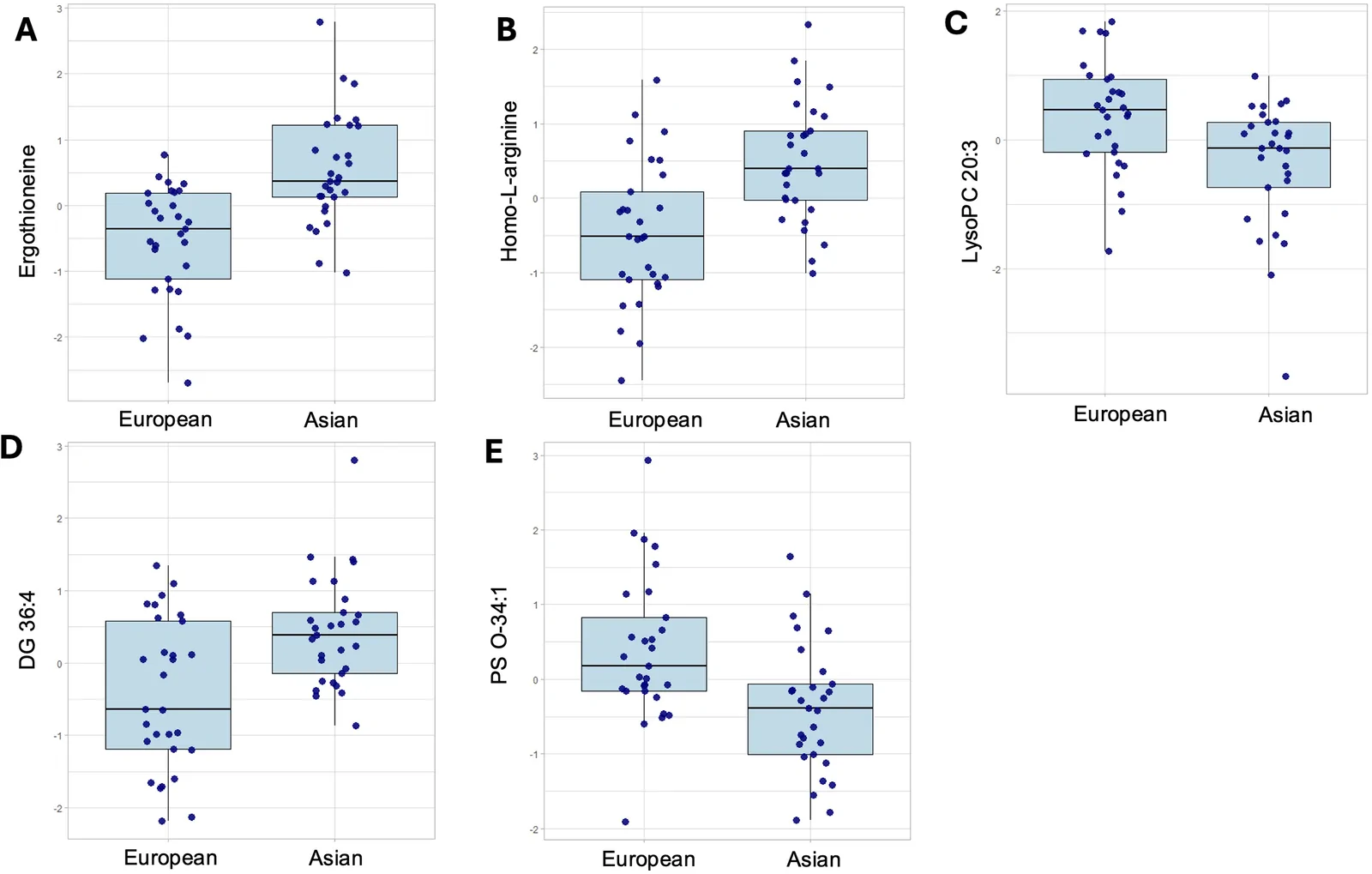
Associations between serum metabolites and self-reported ethnicity. Box-plots of serum abundance levels of **(A)** Ergothioneine, **(B)** Homo-L-arginine, **(C)** LysoPC 20:3, **(D)** DG 36:4, and **(E)** PS O-34:1, in SLE patients of Asian and European ethnicity.

**Table 3:** LASSO-selected serum metabolites predictive of SLE clinical features.

Outcome of interest	Lambda	Accuracy	Balanced accuracy	Metabolites
Asian ethnicity	0.223537	0.6534	0.6534	#
Active disease (SLEDAI-2K > 4)	0.1946656	0.5109	0.5109	#
Active renal disease (SLEDAI-2K proteinuria score > 0)	0.08890966	0.8407	0.7651	**Positive Predictors** • 2-Amino-5-(methylthio)pentanoic acid • 1-Methylhistidine • *N6, N6, N6-trimethyl-L-Lysine* • *Homocitrulline* • Cystine • Glutarylcarnitine **Negative Predictors** • Trans-tetra-dec-2-enoic acid • **5β-cholestane-3α, 7α, 24, 25-tetrol**
Lupus nephritis ever (biopsy-proven)	0.08890966	0.8234	0.8172	**Positive Predictors** • Choline • 1-Pyrroline 2-carboxylic acid • Homocysteine • Uric acid • N6, N6, N6-trimethyl-L-Lysine • *3-Hydroxy-N6, N6, N6-trimethyl-L-Lysine* • Cystine **Negative Predictors** • Homogentisic acid • Hippuric acid • **5β-cholestane-3α, 7α, 24, 25-tetrol** • PS O-34:1 • PI 38:5
Organ damage (SDI >0)	0.0849	0.6583	0.6354	#
LLDAS	0.002124844	0.5521	0.5054	#
Clinical remission (with prednisolone ≤ 5 mg)	0.07742637	0.6988	0.54712	#
Remission off prednisolone	0.1695232	0.7757	0.4998889	**Positive Predictors** • **Glutamic acid**
Prednisolone use	0.1409773	0.6769	0.6618	#
Immunosuppression use	0.0128265	0.7728	0.6698	**Positive Predictors** • Pyruvic.acid • Threonine • Pipecolic.acid • 2-Keto-6-aminocaproate • Allysine • Isopropylmaleate • 2-Amino-5-(methylthio)pentanoic acid • Phenyllactic.acid • Arginine • Salicyluric.acid • Altronate • FA dioxo(10:0) • Cystine • Trimetaphosphoric acid **Negative Predictors** • Succinic.acid • Indoxyl • Formyl-isoglutamine • Ribothymidine • Sucrose • LysoPE 20:5 • LysoPC 18:2 • LysoPC 22:5 • Thyroxine • TG 51:3

# Deemed poor model.

- No predictive metabolite selected.

*Italic*: feature selected by LASSO modeling and independently supported by statistical significance in univariable differential abundance analysis.

Bold: feature selected by LASSO modeling and independently supported by statistical significance in multivariable differential abundance analysis.

Bold and underlined: feature selected by LASSO modeling and independently supported by statistical significance in both univariable and multivariable differential abundance analysis.

Not italic, bold or underlined: feature selected by LASSO modeling and not independently supported by statistical significance in differential abundance analysis.

LLDAS, lupus low disease activity state; SDI, Systemic Lupus International Collaborating Clinics/American College of Rheumatology SLE Damage Index; SLEDAI-2K, SLE Disease Activity Index-2000.

#### Metabolite associations with LN

When investigating renal clinical parameters of interest, we found several clinical associations overlapping between DA and LASSO (**Tables 2**, **3**). Using multivariable DA, a metabolite putatively identified as 5β-Cholestane-3α, 7α, 24, 25-tetrol was negatively associated with active LN (adjusted β -1.51; 95% CI -2.02, -1.00; *p* < 0.001) (**Figure 3A**) and biopsy-proven LN (adjusted β -1.29; 95% CI -1.74, -0.85; *p* < 0.001) (**Figure 4A****),** and was negatively predictive of these parameters using LASSO modelling. Homocitrulline (β 1.15; 95% CI 0.65, 1.64; *p* = 0.004) and N6, N6, N6-trimethyl-L-lysine (β 0.96; 95% CI 0.44, 1.49; p = 0.046) were associated with active LN using univariable DA only (**Figures 3B, C**), and were selected in a predictive LASSO model for this feature. A feature putatively identified as 3-Hydroxy-N6, N6, N6-trimethyl-L-lysine was associated with biopsy-proven LN using univariable DA only (β 0.96; 95% CI 0.49, 1.43; *p* = 0.023) (**Figure 4B**), and was predictive of this feature on LASSO modelling. Homocitrulline was also associated with biopsy-proven LN using multivariable DA (adjusted β 0.94; 95% CI 0.49, 1.39; *p* = 0.022), but was not found to be predictive on LASSO modelling (**Table 3**).

**Figure 3:**
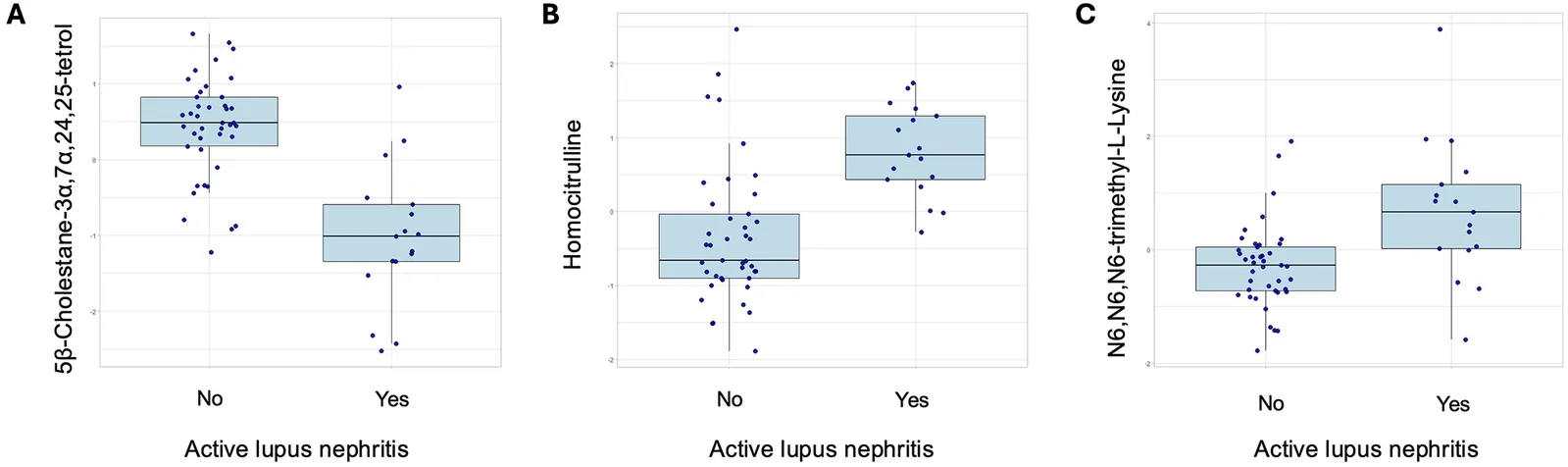
Associations between serum metabolites and active lupus nephritis. Box-plots of serum abundance levels of **(A)** 5β-Cholestane-3α, 7α, 24, 25-tetrol, **(B)** Homocitrulline, and **(C)** N6, N6, N6-trimethyl-L-Lysine, in SLE patients without and with lupus nephritis.

**Figure 4:**
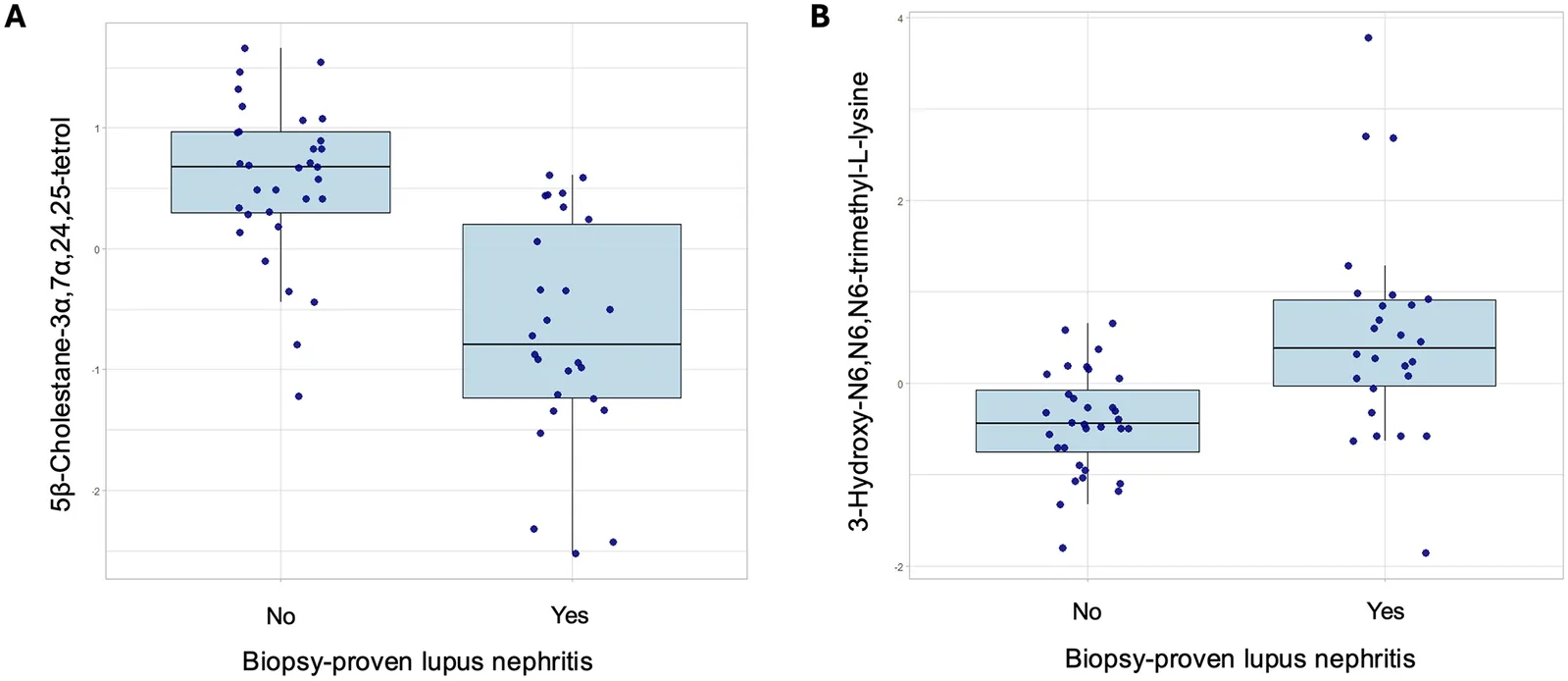
Associations between serum metabolites and active biopsy-proven lupus nephritis. Box-plots of serum abundance levels of **(A)** 5β-Cholestane-3α, 7α, 24, 25-tetrol, and **(B)** 3-Hydroxy-N6, N6, N6-trimethyl-L-lysine, in SLE patients without and with biopsy-proven lupus nephritis.

#### Metabolite associations with overall disease severity

Glutamic acid was associated with glucocorticoid-free remission using multivariable DA (adjusted β 1.20; 95% CI 0.62, 1.78; *p* = 0.048) (**Table 2**; **Figure 5**) and was identified as a candidate predictor of this outcome measure on LASSO modelling (**Table 3**). Of note, no metabolite associations with remission per DORIS criteria were identified.

**Figure 5:**
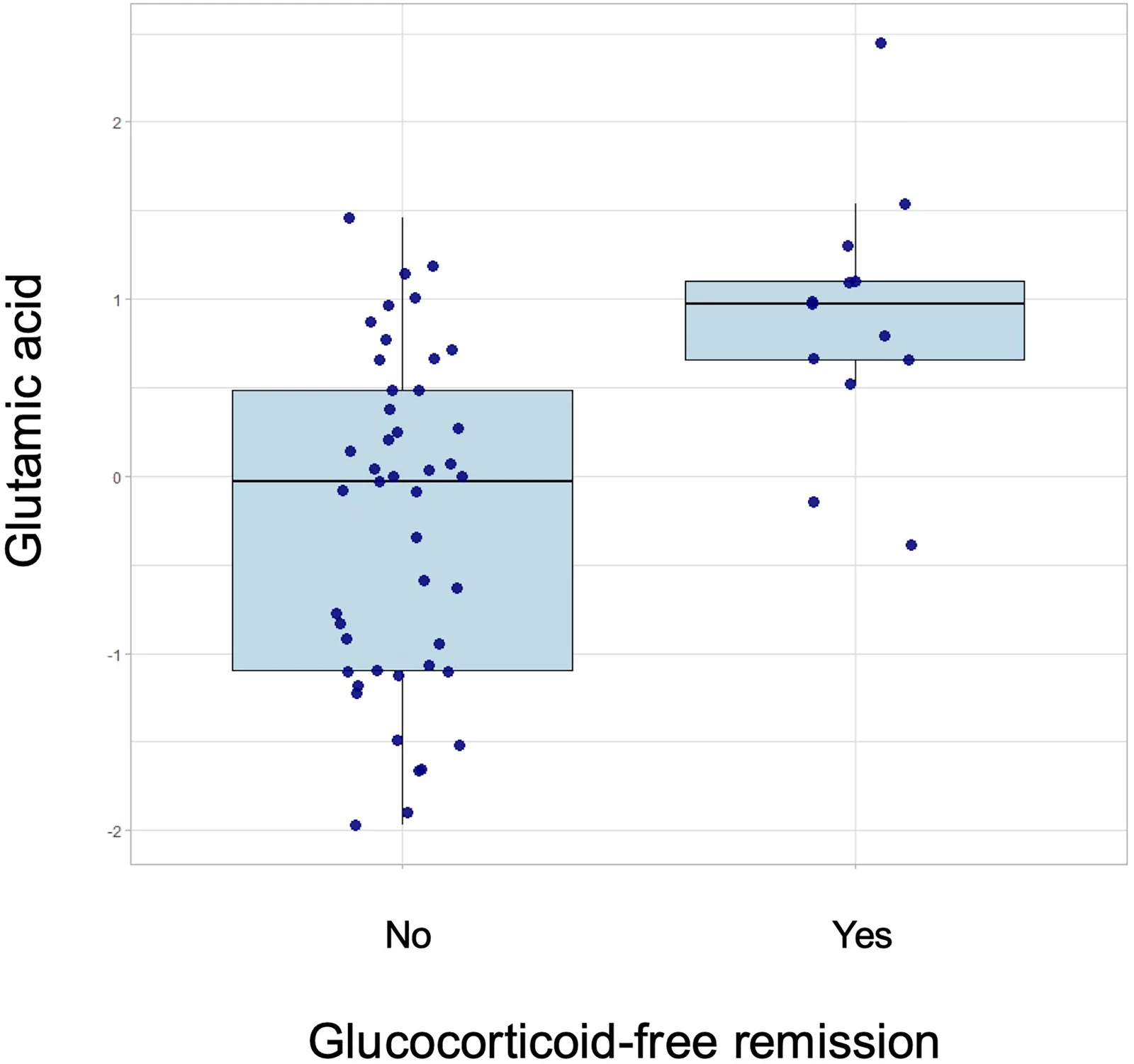
Associations between serum glutamic acid and glucocorticoid-free remission state in SLE. Box-plot of serum abundance levels of glutamic acid in SLE patients without and with glucocorticoid-free remission.

Twenty metabolites were associated with the presence of organ damage using univariable DA (**Table 2**); all attenuated however, after adjustment for age, sex, self-reported ethnicity and renal impairment. We performed a sensitivity analysis refitting the multivariable model, where renal impairment was removed from the adjustment set, and the metabolites present in the univariable model remained attenuated (*data not shown*). No good quality LASSO model for organ damage was fitted (**Table 3**).

#### Metabolite associations with glucocorticoid use

Twenty metabolites were associated with glucocorticoid use using multivariable DA (**Table 2**), although no good quality LASSO model for this feature could be fitted (**Table 3**). Due to the substantial number of metabolites significantly associated with glucocorticoid use, we conducted a sensitivity analysis adding glucocorticoid use to the adjustment set in DA for each clinical parameter of interest. Results of the sensitivity analysis showed consistency with the primary analysis (**Table 2**).

Of note, 24 metabolites were selected by LASSO to predict immunosuppression use (**Table 3**); however, none were associated with this clinical feature in DA (**Table 2**).

MSEA did not identify any significantly enriched pathways for either glucocorticoid use (lowest FDR = 0.15, Histidine Metabolism) or immunosuppression use (lowest FDR = 0.15, Oxidation of Branched Chain Fatty Acids).

### Pathway-level enrichment

To assess whether coordinated, sub-threshold metabolic changes were present across clinical features of interest, MSEA was performed for each clinical feature. This identified significantly enriched pathways for four clinical features, three of which had no individually significant metabolites in multivariable DA: active disease (SLEDAI-2K > 4; Valine, Leucine and Isoleucine Degradation, FDR = 0.0015), organ damage (Citric Acid Cycle and Gluconeogenesis, FDR = 0.043 each), and clinical remission per DORIS criteria (Phenylalanine and Tyrosine Metabolism, FDR = 0.028; Valine, Leucine and Isoleucine Degradation, FDR = 0.041). For remission off prednisolone, four pathways were enriched (Urea Cycle, Tryptophan Metabolism, Purine Metabolism, and Glutamate Metabolism; FDR = 0.044 each), including Glutamate Metabolism, consistent with glutamic acid being the sole individually significant metabolite for this feature (**Table 2**). Full pathway-level results for all ten clinical features are provided in **Supplementary Table 4**.

### Metabolite identity validation

Of the above-described metabolites, most were identified by direct comparison of their retention times with authentic standards. There were two overlapping associations between DA and LASSO for which metabolites were not identified with certainty; 3-hydroxy-N6, N6, N6-trimethyl-L-lysine and 5β-Cholestane-3α, 7α, 24, 25-tetrol.

#### 3-Hydroxy-N6, N6, N6-trimethyl-L-lysine

There was no standard available for 3-Hydroxy-N6, N6, N6-trimethyl-L-lysine in the standard library but we note that MS/MS of the feature putatively identified as 3-Hydroxy-N6, N6, N6-trimethyl-L-lysine in PQC yields a spectrum, which is essentially consistent with this structure. In detail, the major product ions can be assigned as protonated trimethylamine, the loss of trimethylamine, the combined loss of trimethyl amine and water, the combined loss of trimethyl amine, water and carbon monoxide (**Supplementary Figure 1S**).

#### 5β-Cholestane-3α, 7α, 24, 25-tetrol

The precise assignment of the feature putatively identified as 5β-Cholestane-3α, 7α, 24, 25-tetrol is more speculative. IDEOM assigns this feature as the formate adduct of a metabolite with the molecular formula C_27_H_48_O_4_. An alternative assignment would be a carbonate adduct with the molecular formula C_27_H_48_O_3_. This likely corresponds to a tetra- or tri-hydroxylated cholestane structure respectively, of which there are several theoretical possibilities. For example, for both molecular formulas there are several entries in the LIPID MAPS structural database (32, 33) in either the cholesterol and derivatives [ST0101] or C27 bile acids, alcohols, and derivatives [ST0403] subclasses. Consequently, we consider that at this stage this feature is almost certainly an oxidized cholesterol derivative; however, we remain uncertain of its exact structure.

## Discussion

Untargeted serum metabolomics is emerging as a promising method of biomarker discovery in SLE. However, to date, there have been few studies investigating associations between serum metabolomic profiles and detailed SLE clinical phenotypes, including validated disease outcome measures. In this study, we generated untargeted serum MS metabolomic profiles in a highly clinically annotated cohort of 60 patients with SLE, and explored associations with disease phenotypes, including validated outcome measures.

Using unsupervised clustering analysis, we demonstrated that our patient cohort could be divided into three clusters based on metabolomic profiles, including a cluster of patients less likely to have organ damage and to use prednisolone, and a cluster of patients more likely to have active lupus nephritis, renal impairment and organ damage.

When exploring associations between metabolites and clinical features of interest, we found several overlapping associations between DA and LASSO, including 5β-Cholestane-3α, 7α, 24, 25-tetrol which was negatively associated with both active and biopsy-proven LN, homocitrulline and N6, N6, N6-trimethyl-L-lysine, both of which were positively associated with active LN, 3-Hydroxy-N6, N6, N6-trimethyl-L-lysine which was positively associated with biopsy-proven LN, and glutamic acid, which was positively associated with glucocorticoid-free remission. We also found 20 metabolites associated with glucocorticoid use on multivariable DA, although no predictive LASSO model for glucocorticoid use could be generated.

Our finding of a metabolite profile-based patient cluster characterised by lower levels of organ damage and lower rates of prednisolone use is in keeping with the strong association between organ damage and prednisolone use described in the literature (34). A separate patient cluster was associated with significantly increased rates of active LN, renal impairment and organ damage, and numerically higher rates of biopsy-proven LN and disease activity according to SLEDAI-2K. Although patient numbers in this cluster were small, our findings support the potential for serum metabolomic profiling to identify patients with more severe SLE and renal involvement. Studies investigating whether serum metabolite profiles can predict which patients are more likely to develop severe disease, LN and organ damage, and whether metabolite levels fluctuate according to overall or renal disease activity, and LN class would be of interest.

To further characterise the biological basis of this cluster structure, we ranked all metabolites by their cos^2^ value on the first two principal components, a measure of how strongly each metabolite’s variance is captured by the components underlying the observed clustering, independent of the cluster assignments themselves. Briefly, the three clusters comprised a low-disease-burden group, characterised by lower organ damage and prednisolone use; a high-burden group, characterised by active LN, renal impairment and organ damage; and an intermediate group. The metabolites with the highest cos^2^, including alpha- and beta-D-glucose, imidazoleacetic acid riboside, N-formimino-L-glutamate, 2, 3, 4, 5-tetrahydroxypentanoic acid, 2, 3-dihydroxy-isovalerate, and homocitrulline, were specifically elevated in the high-burden group, rather than varying incrementally across all three clusters in proportion to overall disease burden. Notably, alpha-D-glucose is a member of the Gluconeogenesis pathway, which was independently identified as significantly enriched for organ damage in our metabolite set enrichment analysis (*see* Metabolite set enrichment analysis), providing convergent evidence across two analytical approaches for a role of glucose/energy metabolism in this patient subgroup. Together, these findings support the interpretation that this cluster may capture a metabolically distinct patient subgroup, rather than simply representing a disease-severity gradient, although validation in an independent cohort and mechanistic studies would be required before these clusters can be considered established metabolic endotypes.

The increased N6, N6, N6-trimethyl-L-lysine in active LN, and increased 3-Hydroxy-N6, N6, N6-trimethyl-L-lysine in biopsy-proven LN indicate alterations in carnitine biosynthesis and fatty acid oxidation. N6, N6, N6-trimethyl-L-lysine is a methylated derivative of the amino acid lysine, and is part of the carnitine biosynthesis pathway, and a coenzyme of fatty acid oxidation (35). It is associated with inflammatory conditions including atherosclerosis (36). Interestingly, however, it has also been shown to have a negative association with ankylosing spondylitis (37), but has not previously been described in association with SLE or inflammatory chronic kidney disease. 3-Hydroxy-N6, N6, N6-trimethyl-L-lysine is the 3-hydroxy derivative of N6, N6, N6-trimethyl-L-lysine and is also involved in the carnitine biosynthesis pathway (35). To our knowledge, it has not previously been described in association with SLE or other autoimmune diseases.

Consistent with this increase in the methylated derivatives of lysine we observed in LN, we also detected an increase in another lysine derivative, generated by carbamylation, in LN. We report a novel positive association between homocitrulline and active LN using univariable DA and LASSO, as well as with organ damage using univariable DA only. Homocitrulline is an L-alpha amino acid which is physiologically non-essential (35), although it is reported to be associated with an increased risk of renal failure (38). Whilst it is possible that the association we observed between homocitrulline and organ damage was due to its known association with renal impairment, given the association was attenuated after adjusting for renal impairment, the association that we discovered with active LN persisted despite adjustment for this covariate. To our knowledge, elevated levels of homocitrulline have not previously been described in SLE or LN. Antibodies against both citrullinated and homocitrullinated proteins are known to contribute to the pathogenesis of RA (39), and homocitrulline levels are elevated in the synovial tissue and rheumatoid nodules of patients with seropositive RA, compared to those with seronegative RA or osteoarthritis (40). Elevated serum levels of homocitrulline have also been described in patients with Behçet’s disease (41). While autoantibodies against homocitrullinated proteins have been described in patients with SLE (42, 43), this has not been shown consistently, with other studies suggesting that these antibodies are specific to patients with RA (44). These results underscore the need for larger studies to better define the role of homocitrulline and homocitrullinated proteins in SLE, enabling stratification by ISN/RPS (International Society of Nephrology/Renal Pathology Society) LN class. Interestingly, all three metabolites associated with LN, homocitrulline, N6, N6, N6-trimethyl-L-lysine and 3-hydroxy-N6, N6, N6-trimethyl-L-lysine, are lysine derivatives. Homocitrulline (ϵ-carbamyllysine), a hallmark product of protein carbamylation, a non-enzymatic post-translational modification resulting from the reaction of cyanate with lysine residues, is of particular interest, as its formation is enhanced by both uremia and inflammatory processes and has been increasingly linked to chronic kidney disease and adverse renal outcomes (38, 45). Collectively, these findings suggest that altered lysine metabolism and post-translational modification pathways, including protein carbamylation, may be reflected in the metabolomic signature of LN.

We found a novel negative association between a metabolomic feature putatively identified as 5β-Cholestane-3α, 7α, 24, 25-tetrol and both active and biopsy-proven LN. 5β-Cholestane-3α, 7α, 24, 25-tetrol is an intermediate metabolite in the bile acid synthetic pathway (35), but to our knowledge, has not previously been described in association with SLE. Cholestane-3β, 5α, 6β-triol is an alternative candidate assignment for this feature, and has previously been demonstrated to be more highly expressed in murine SLE skin lesions compared with healthy skin, along with other metabolites involved in oxidised cholesterol metabolism (46). These metabolites are hypothesised to be up-regulated by fibroblasts present in SLE skin lesions, and in turn to promote the recruitment of pro-inflammatory immune cells to skin lesions (46). These metabolites suggest cholesterol and bile acid involvement in SLE pathogenesis, which should be further investigated. Our finding of four metabolites associated with LN highlights the potential for serum metabolomics to yield organ-specific SLE biomarkers, which could provide complementary information beyond currently used, non-organ specific serological biomarkers, such as anti-dsDNA, complement, and non-SLE specific ones, such as proteinuria and haematuria.

To our knowledge, this is the first study to report an association between glutamic acid and glucocorticoid-free remission in patients with SLE, although glutamic acid has previously been studied in both human SLE and mouse models (47, 48). A study by Zhang X et al. demonstrated that glutamic acid levels were significantly elevated in the splenic mononuclear cells of MRL/lpr mice and the peripheral blood mononuclear cells of patients with SLE (48). They also found that inhibiting the conversion of glutamine to glutamic acid improved LN in MRL/lpr mice (48). This was hypothesised to be due to suppression of pro-inflammatory Th17 and follicular T cells, which decreased in number in response to inhibition of glutamine metabolism (48). A further metabolomics study in SLE patients found that serum glutamic acid was significantly positively associated with SLEDAI-2K and negatively associated with C4 levels (47). This is out of keeping with our finding of a positive association between glutamic acid and glucocorticoid-free remission, suggesting the need for further studies to clarify the relationship between glutamic acid and disease activity in SLE, and further explore the underlying pathophysiological mechanisms driving observed phenotypic associations.

We identified 20 metabolites associated with prednisolone use on multivariable DA, highlighting the potential for glucocorticoid use to confound results in studies of serum metabolomics in SLE. This finding is in keeping with several studies which have demonstrated that both rats and healthy human subjects treated with dexamethasone have significant differences in metabolite profiles compared to those who were not (49–51). Interestingly however, adjustment for glucocorticoid use in our patient cohort did not markedly change our results. While other studies have also found metabolites associated with glucocorticoid use in SLE patients (52, 53), none of these have examined the impact of adjusting for glucocorticoid use on associations between clinical phenotypes and serum metabolomic profiles in patients with SLE (52, 53). MSEA did not identify any significantly enriched pathways for glucocorticoid use, despite the substantial number of individually significant metabolites associated with glucocorticoid use, suggesting these associations are not sufficiently concentrated within a single coordinated metabolic pathway.

MSEA provided a complementary, systems-level view of these clinical associations. Notably, active disease, organ damage, and clinical remission per DORIS criteria showed no individually significant metabolite associations in multivariable DA, yet each was associated with significant enrichment of specific metabolic pathways: The Valine, Leucine and Isoleucine Degradation pathway was enriched for active disease, reflecting perturbation of branched-chain amino acid catabolism, rather than a direct increase in degradation; central energy metabolism (the Citric Acid Cycle and Gluconeogenesis pathways) was enriched for organ damage, and aromatic and branched-chain amino acid catabolism for clinical remission. This suggests that for these clinical features, disease-associated metabolic perturbation may be distributed as small, coordinated shifts across many metabolites within a pathway rather than concentrated in one or two metabolites large enough to reach significance individually, a pattern individual-metabolite DA is structurally underpowered to detect. For remission off prednisolone, pathway-level enrichment of glutamate metabolism was consistent with the individual finding of glutamic acid. Branched-chain amino acid catabolism has been shown to sustain interferon-driven immune cell activation in autoimmune arthritis (54), while central energy metabolism (glycolysis and mitochondrial oxidative metabolism) has been shown to drive CD4+ T cell effector function in murine lupus model (55), and their enrichment reported here may reflect altered immunometabolic demand associated with disease activity and organ damage accrual in SLE; however, given the exploratory nature of this analysis, these pathway-level associations should be regarded as hypothesis-generating rather than confirmatory.

Our study had several limitations. Albeit excluding metabolites with missing values reduced metabolome coverage, this filtering strategy maximised analytical robustness in the context of a discovery-orientated approach with a modest sample size. While we selected only metabolites that were identified with relatively high levels of confidence to be included in the final dataset, there was still a degree of uncertainty regarding the exact identity of several metabolites for which we found associations with clinical features. In particular, the chemical identity of the putatively identified metabolites 3-Hydroxy-N6, N6, N6-trimethyl-L-lysine and 5β-Cholestane-3α, 7α, 24, 25-tetrol needs to be validated in future work. This will involve additional LC-MS analysis against authentic standards of candidate structures compared to a subset from the analysis known to have either high or low levels of these features. Similarly, our MSEA was limited by incomplete mapping of platform-specific metabolite identifiers to curated pathway databases (75.5% of metabolites mapped successfully), meaning some coordinated changes (particularly among putatively identified or chain-specific lipid species) may not have been captured; these pathway-level findings should therefore be considered exploratory. Our patient cohort size, whilst comparable to most other metabolomics studies in SLE, was modest, which may have rendered our study under-powered to find additional statistically significant associations between metabolites and clinical features, particularly in the clustering analysis, in which patient numbers in each cluster were small. Our modest cohort size also limited our ability to adjust for factors which may have influenced metabolite profiles in our multivariable models. Furthermore, due to our modest cohort size, we focused on metabolites which were associated with clinical features of interest using both DA and LASSO, to ensure that our major reported findings were as statistically robust as possible. However, it would be of interest to validate other metabolites associated with clinical features of interest using DA, or selected in LASSO models in independent patient cohorts, to facilitate the discovery of multi-metabolite signatures associated with SLE phenotypes. Externally validated parsimonious multi-metabolite panels may offer advantages over individual biomarkers by more comprehensively capturing the biological heterogeneity underlying distinct clinical phenotypes and outcomes in SLE, while remaining practical for clinical implementation.

The single-centre nature of our study, as well as the intentional, non-random approach to participant selection used to construct a well-balanced cohort, may have predisposed the study to inherent selection bias and could limit the extent to which these findings are generalisable to the wider SLE population. In particular, our finding of five metabolites associated with Asian ethnicity, and 20 metabolites associated with glucocorticoid use, highlight the possibility of differences in metabolomic profiles in patient cohorts of different ethnicities and exposed to different treatment regimes than ours. While we did adjust for confounders which we thought were most likely to influence metabolomic profiles in our patients, including renal impairment and glucocorticoid use, a larger cohort size may have allowed adjustment for additional potential confounders, including medications other than glucocorticoids, particularly hydroxychloroquine and individual immunosuppressive agents, as well as medical co-morbidities other than renal impairment. In addition, although it is known that fasting status and diet influence metabolomic profiles (56, 57), we did not have dietary information or information regarding the fasting status of patients in our study. While our clinical data was highly detailed, there was a moderate median time difference between serum study collection and assessment of organ damage, which may have influenced results with respect to organ damage, given this can accrue rapidly in some patients with SLE.

Finally, in our study, metabolomic profiles were assessed at a single point in time. However, metabolites fluctuate with changes in physiological state, and metabolomic profiles therefore only provide a snapshot of what is occurring at a cellular level at a particular time-point. While this makes cross-sectional clinical associations with metabolite profiles interesting and clinically relevant, future studies exploring whether changes in metabolite levels at various time points correspond to changes in clinical state in SLE are needed prior to determining whether the candidate metabolites identified in this study would be most suitable as diagnostic, prognostic, disease-monitoring or treatment-response biomarkers. Future studies of urinary metabolomics in patients with LN would also be of interest to validate our metabolite associations with LN, given the anatomical proximity of the urinary matrix to renal tissue.

In conclusion, we generated untargeted serum metabolomic profiles in SLE patients using LC-MS. Using unsupervised clustering analysis, we identified a patient cluster with metabolite profiles associated with active LN, renal impairment and organ damage, and another cluster associated with lower rates of organ damage and prednisolone use. We identified 20 individual metabolites associated with glucocorticoid use, highlighting the importance of accounting for this in metabolomics studies in SLE. We identified novel metabolite associations with LN, particularly methylated and carbamylated lysine derivatives, and glucocorticoid-free remission, demonstrating the potential of serum metabolomics to yield clinically useful biomarkers. Further studies of serum metabolomics in SLE patients to validate and extend these findings are warranted.

## Data Availability

The study findings and supporting materials are included in the article/**Supplementary Material**. Access to the underlying data is restricted due to ethical and patient consent requirements. Further inquiries can be directed to the corresponding author.
