# Supplementary material for: Untargeted serum metabolomics in systemic lupus erythematosus reveals candidate metabolomic signatures associated with clinical phenotypes and glucocorticoid use
Source: Front Immunol. 2026 Oct 6;17:1925827. doi: 10.3389/fimmu.2026.1925827 (PMC13680200; doi:10.3389/fimmu.2026.1925827)
Supplement: Supplementary file 1 [file SupplementaryFile1.docx]

**Supplementary Figure 1. The MS/MS spectra corresponding to the feature putatively identified as 3-Hydroxy-N6,N6,N6-trimethyl-L-lysine in the PQC sample.**

Although not definitive proof of the structure it is noteworthy that the major product ions can be assigned with fragments consistent with this identification.
