# Supplementary material for: Untargeted serum metabolomics in systemic lupus erythematosus reveals candidate metabolomic signatures associated with clinical phenotypes and glucocorticoid use
Source: Front Immunol. 2026 Oct 6;17:1925827. doi: 10.3389/fimmu.2026.1925827 (PMC13680200; doi:10.3389/fimmu.2026.1925827)
Supplement: Supplementary file 2 [file Table1.docx]

**Supplementary table 1.** **Clinical data collected.**

| **Data extracted from ALRB database** | **Definitions** | **Ref** |
| --- | --- | --- |
| *Enrolment visit* | | |
| Date of birth | - | - |
| Sex | - | - |
| Ethnicity | Self-reported Asian or European | - |
| Paid employment | - | - |
| Receipt of disability pension | - | - |
| Household income | Binarised to < $35,000 AUD *vs* ≥ $35,000 AUD per annum | - |
| Education level | Binarised to below secondary level *vs* secondary level or above | - |
| Smoking status | Current, past or never smoker | - |
| Significant alcohol consumption | Binarised to > 10 *vs* ≤ 10 standard drinks per week | - |
| *Visit closest to study sample collection* | | |
| Disease duration | Calculated by subtracting date of SLE diagnosis from date of study serum sample collection | - |
| SLEDAI-2K | Active disease defined as SLEDAI-2K > 4  Highly active disease defined as SLEDAI-2K ≥ 10 | (1)  (2) |
| Renal disease activity | Presence of SLEDAI-2K proteinuria (+/- other renal SLEDAI-2K parameters) | - |
| Flare | As determined by SELENA-SLEDAI flare index (SFI) | (3) |
| Low disease activity | Attainment of LLDAS | (4) |
| DORIS remission | Attainment of DORIS remission criteria | (5) |
| Glucocorticoid free remission | Attainment of DORIS remission criteria but not allowing glucocorticoid use | - |
| Prednisolone use and dose | Prednisolone use (Y/N) and dose | - |
| Hydroxychloroquine use | Hydroxychloroquine use (Y/N) | - |
| Immunosuppression use | Including methotrexate, mycophenolate, azathioprine, leflunomide, tacrolimus, rituximab, belimumab, baricitinib and abatacept. | - |
| ESR (mm/hr) | - |  |
| Renal function | eGFR (mL/min/1.73m^2^) |  |
| *Annual visit closest to study sample collection* | | |
| Organ damage | SDI > 0 (SDI assessed at annual visit closest to serum study sample collection; median (IQR) time between organ damage assessment and serum study sample collection 98 (56, 154) days) | (6) |

AMS (adjusted mean SLEDAI-2K), AUD (Australian dollars), CNS (central nervous system), DORIS (Definition of Remission in SLE), eGFR (estimated glomerular filtration rate), ESR (erythrocyte sedimentation rate), IQR (inter-quartile range), LLDAS (lupus low disease activity state), LN (lupus nephritis), SDI (Systemic Lupus International Collaborating Clinics/American College of Rheumatology Damage Index), SLEDAI-2K (SLE Disease Activity Index-2000)

(Adapted from Eades *et al. (7))*

1.	Vincent FB, Kandane-Rathnayake R, Koelmeyer R, Harris J, Hoi AY, Mackay F, et al. Associations of serum soluble Fas and Fas ligand (FasL) with outcomes in systemic lupus erythematosus. Lupus Sci Med. 2020;7(1).

2.	Koelmeyer R, Nim HT, Nikpour M, Sun YB, Kao A, Guenther O, et al. High disease activity status suggests more severe disease and damage accrual in systemic lupus erythematosus. Lupus Sci Med. 2020;7(1).

3.	Buyon JP, Petri MA, Kim MY, Kalunian KC, Grossman J, Hahn BH, et al. The effect of combined estrogen and progesterone hormone replacement therapy on disease activity in systemic lupus erythematosus: a randomized trial. Ann Intern Med. 2005;142(12 Pt 1):953-62.

4.	Franklyn K, Lau CS, Navarra SV, Louthrenoo W, Lateef A, Hamijoyo L, et al. Definition and initial validation of a Lupus Low Disease Activity State (LLDAS). Annals of the Rheumatic Diseases. 2016;75(9):1615.

5.	van Vollenhoven RF, Bertsias G, Doria A, Isenberg D, Morand E, Petri MA, et al. 2021 DORIS definition of remission in SLE: final recommendations from an international task force. Lupus Sci Med. 2021;8(1).

6.	Stoll T, Seifert B, Isenberg DA. SLICC/ACR Damage Index is valid, and renal and pulmonary organ scores are predictors of severe outcome in patients with systemic lupus erythematosus. Br J Rheumatol. 1996;35(3):248-54.

7.	Eades LE, Papadaki A, Lennard K, Hoi AY, Kandane-Rathnayake R, Morand EF, et al. Serum IgA autoantibody profiling in systemic lupus erythematosus reveals associations with disease phenotypes. RMD Open. 2026;12(3):e006811.
