# Supplementary material for: Untargeted serum metabolomics in systemic lupus erythematosus reveals candidate metabolomic signatures associated with clinical phenotypes and glucocorticoid use
Source: Front Immunol. 2026 Oct 6;17:1925827. doi: 10.3389/fimmu.2026.1925827 (PMC13680200; doi:10.3389/fimmu.2026.1925827)
Supplement: Supplementary file 3 [file Table2.docx]

**Supplementary Table 2. Descriptive statistics of metabolomic-based SLE patient clusters.**

| ***Clinical variable of interest*** |  | **Cluster 1**  **n = 32** | **Cluster 2**  **n = 20** | **Cluster 3**  **n = 5** |
| --- | --- | --- | --- | --- |
| **Age (years)***, median (IQR)* |  | 37.5 (30.75, 48.5) | 51 (43.0, 57.75) | 36 (35, 41) |
| **Female sex***, n (%)* |  | 30 (94) | 19 (95) | 3 (60) |
| **Asian ethnicity**^*^*, n (%)* |  | 19 (59) | 7 (35) | 3 (60) |
| **Active disease (SLEDAI-2K > 4)***, n (%)* |  | 15 (47) | 9 (45) | 4 (80) |
| **Active renal disease (SLEDAI-2K proteinuria >0^1^)***, n (%)* |  | 7 (22) | 5 (25) | 4 (80) |
| **Biopsy-proven LN***, n (%)* |  | 12 (38) | 9 (45) | 4 (80) |
| **Organ damage (SDI > 0***), n (%)* |  | 10 (33) ^^^ | 16 (89) ^^^ | 5 (100) |
| **Low disease activity (LLDAS)***, n (%)* |  | 12 (38) | 9 (47) ^#^ | 0 (0) |
| **DORIS remission***, n (%)* |  | 10 (31) | 7 (35) | 0 (0) |
| **Remission off glucocorticoids***, n (%)* |  | 8 (25) | 5 (25) | 0 (0) |
| **Renal impairment (eGFR <60 ml/min/1.73m^2^)***, n (%)* |  | 0 (0) | 1 (5) | 4 (80) |
| **Current glucocorticoids use***, n (%)* |  | 10 (31) | 13 (65) | 3 (60) |
| **Current immunosuppressive use***, n (%)* |  | 25 (78) | 14 (70) | 4 (80) |

^*^ All patients not of Asian ethnicity were of European ethnicity.

^1^ Proteinuria > 0.5g/g or 0.05g/mmol or 0.5g/24hr

^#^ Data missing for 1 patient, ^^^ data missing for 2 patients

DORIS, Definition of Remission in SLE; eGFR, estimated glomerular filtration rate; IQR (interquartile range), LLDAS (lupus low disease activity state), SDI (Systemic Lupus International Collaborating Clinics/American College of Rheumatology SLE Damage Index), SLE (systemic lupus erythematosus), SLEDAI-2K (SLE Disease Activity Index-2000)
