# Supplementary material for: Untargeted serum metabolomics in systemic lupus erythematosus reveals candidate metabolomic signatures associated with clinical phenotypes and glucocorticoid use
Source: Front Immunol. 2026 Oct 6;17:1925827. doi: 10.3389/fimmu.2026.1925827 (PMC13680200; doi:10.3389/fimmu.2026.1925827)
Supplement: Supplementary file 4 [file Table3.docx]

**Supplementary Table 3. Ranking of serum metabolite features by contribution to the principal components underlying patient cluster separation (Figure 1B).**

cos² (PC1+PC2): combined squared cosine value for PC1 and PC2.
