# Supplementary material for: Untargeted serum metabolomics in systemic lupus erythematosus reveals candidate metabolomic signatures associated with clinical phenotypes and glucocorticoid use
Source: Front Immunol. 2026 Oct 6;17:1925827. doi: 10.3389/fimmu.2026.1925827 (PMC13680200; doi:10.3389/fimmu.2026.1925827)
Supplement: Supplementary file 5 [file Table4.docx]

**Supplementary Table 4.** **Metabolite set enrichment analysis (MSEA) results for the ten clinical features assessed by differential abundance analysis (Table 2).**

Exposure: the clinical feature of interest

Pathway: name of the SMPDB metabolite set (metabolic pathway) tested for enrichment.

Size: refers to the number of metabolites in the metabolite set (pathway) present in the ranked list.

NES: Normalized Enrichment Score; positive values indicate pathway metabolites are shifted toward higher t-statistics for that clinical feature, negative values the reverse

pval: unadjusted (nominal) *p*-value from the enrichment permutation test.

padj: Benjamini-Hochberg false discovery rate (FDR)-adjusted *p*-value, corrected within each clinical feature's set of tested pathways.
